# The complexity of physician-patient communication and its impact in non-medical fields. A surgical oncology approach

**DOI:** 10.25122/jml-2023-0154

**Published:** 2023-04

**Authors:** Ciprian Ianovici, Victor Lorin Purcărea, Iuliana-Raluca Gheorghe, Alexandru Blidaru

**Affiliations:** 1 Department of Health Care Marketing and Medical Technology, Carol Davila University of Medicine and Pharmacy, Bucharest, Romania; 2 Department of Surgical Oncology, Carol Davila University of Medicine and Pharmacy, Bucharest, Romania

**Keywords:** physician-patient communication, surgical oncology, quality of health care services, image

## Abstract

Physician-patient communication is essential for determining high-quality healthcare, as this may influence patients’ satisfaction with care, their understanding of medical information, coping skills specific to a disease, and raise treatment adherence. In the field of surgical oncology, most healthcare communication develops around the disease, treatment, and healthcare planning, overlooking psychological functioning and patients’ well-being. To address this issue and avoid unmet patient needs, patient-centered communication requires specific skills designed to enable physicians to identify, acknowledge and respond to patients’ thoughts and feelings over an extended period. The aim of this study was to investigate the integration of patient-physician communication in a non-medical system made up of patient-physician communication, perceived healthcare quality, and the image of a physician or a healthcare organization with a specific focus on surgical oncology. The sample comprised 157 breast cancer patients who reported highly satisfactory levels of perceived communication skills of physicians and the quality of services. Moreover, patients expressed their willingness to recommend these physicians to their family and friends, which further contributes to the positive image of physicians. Nevertheless, it is important to emphasize the ongoing need for continuous attention to the communication skills of surgical oncologists, as each cancer patient's experience is unique and necessitates a personalized form of interaction.

## INTRODUCTION

Despite the increased availability of healthcare information for cancer patients in recent years, accessing timely and relevant information has become progressively challenging, particularly when it does not come directly from their physicians [1]. However, the emergence of the internet as a source of healthcare information has offered the possibility of exchanging and supporting cancer patients with specific information, creating an interactive environment without geographical barriers [2]. Empowering patients with specific information has proven beneficial, particularly in guiding them to ask pertinent questions during consultations [3]. Nonetheless, this empowerment can strain patient-physician communication, as patients may express doubt and distrust based on online information, potentially threatening the authority of physicians [4]. Moreover, poor communication between patients and physicians has been linked to negative outcomes, including patient uncertainty, denial, anxiety, depression, and difficulties adapting to long-term treatment [5]. Thus, effective communication may influence patient satisfaction, facilitating comprehension of medical information, promoting treatment adherence [6], and mitigating cognitive dissonance when seeking second opinions or recommendations online [7].

In practice, few surgical oncologists or nurses have received formal education in communication skills using methods that should trigger change, confidence, and competence [8,9]. In fact, some surgical oncologists acknowledged that insufficient communication and management skills contribute significantly to heightened stress levels, job dissatisfaction, and emotional burden [10]. For instance, scientific literature has underscored the psychological impact of decision-making in breast cancer surgery and emphasized the importance of effective communication in establishing accurate diagnoses and treatment options [11]. Furthermore, this research demonstrated the significance of providing timely, accurate, and clear information during decision-making, ultimately shaping the patient's role—whether passive, active, or collaborative [12]. While patients' needs and preferences may vary, physicians are responsible for providing comprehensive information regarding diagnosis, prognosis, treatment options, and emotional and physical support [13].

Although physicians have good communication skills, they often struggle with time constraints and poor continuity of care [14,15], leaving patients with unmet needs and unaddressed symptoms and coping strategies, particularly in terms of psychosocial support [16]. Responding to the emotional aspects of communication with cancer patients can be particularly challenging for doctors, sometimes leading to overwhelming consultations [17].

Patient-physician communication is essential in everyday interactive activity, especially in providing high-quality healthcare services [18]. Delivering high-quality healthcare services contributes to patients' perceived quality of care. According to Grönroos, healthcare quality encompasses clinical aspects, which focus on the accuracy of medical diagnoses and procedures, and functional aspects, which pertain to how services are delivered to patients [19]. Therefore, consumers often struggle to distinguish between curing and caring performances [20]. Curing performance refers to the technical quality of healthcare while caring performance encompasses service attributes and environmental facilities, such as cleanliness and the attitudes of personnel. In fact, during an interactive consultation, a healthcare service is perceived as properly provided when patients’ perceptions are met or exceeded [21]. The communication process is part of both the curing and caring activities, although they differ in their aims. Communication processes embedded in treatment-related elements constitute curing activities, while those incorporating prevention information align with caring activities.

**Figure 1:**
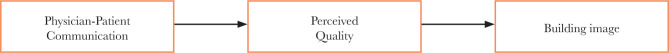
Theoretical framework

Perceived quality of care significantly influences word-of-mouth communication, such as recommendations, and plays a substantial role in shaping the image of physicians and healthcare organizations [22]. The image consists of two dimensions - past vs./ present feelings, beliefs, and attitudes of the patients and present feelings, beliefs, and attitudes [23]. A growing body of research and guidelines supports the idea that physicians do not need to possess inherent excellent communication skills but rather learn to communicate effectively during medical interactions with patients [24]. Therefore, the underlying scope of this study is to explore patient-physician communication as an antecedent of perceived healthcare quality, which may contribute to image building. Specifically, this research aimed to investigate patient-physician communication in surgical oncology and integrate it into a system, along with perceived healthcare quality and image (Figure 1).

The research focused on achieving the following objectives:

1. Identify the socio-demographic profile of patients admitted to the surgical oncology department of a hospital in Bucharest, Romania;
2. Evaluate the perceived quality of care among patients at two different time points;
3. Determine the quality of physician-patient communication based on the perspectives of the patients;
4. Assess patients’ intention to recommend healthcare services to their family and friends.

## Material and methods

The study employed a cross-sectional design and utilized convenience sampling to select 157 patients diagnosed with breast cancer and hospitalized in the surgical oncology department of a healthcare organization in Bucharest, Romania.

Data collection was conducted using a self-administered questionnaire consisting of two sections. The first section focused on capturing the socio-demographic profile of the patients, while the second section encompassed specific items related to the perceived quality of physician-patient communication.

To ensure the confidentiality and anonymity of the participants, appropriate measures were taken during the data collection process. Patients were fully informed about the study's objectives and provided their informed consent by signing an approved consent form.

Data analysis was carried out using IBM Statistics version 25. Descriptive statistics, including absolute values and percentages, were employed to describe the qualitative data. For analyzing the relationship between socio-demographic variables and the communication process, Fisher's exact test was utilized. Furthermore, Z-tests with Bonferroni corrections were performed for a more accurate overview.

## Results

Most of the patients included in the study were women (96.20%) due to the gender-specific nature of breast cancer. The highest proportion of patients fell within the age groups of 48-53 years (51; 32.5%) and 54-59 years (50; 31.8%) (Table 1).

**Table 1:** The distribution of patients according to age.

Age	Frequency	Percent
**<18 years**	2	1.3%
**18–23 years**	4	2.5%
**24–29 years**	4	2.5%
**30–35 years**	4	2.5%
**36–41 years**	9	5.7%
**42–47 years**	14	8.9%
**48–53 years**	51	32.5%
**54–59 years**	50	31.8%
**≥60 years**	19	12.1%

During the hospitalization period, most patients requested to be supervised by a nominal physician (137; 87.3%), and 11 (7.00%) patients asked for second opinions regarding their diagnosis.

In what concerns the perceived healthcare quality, most patients were satisfied (81; 51.6%) or very satisfied (74; 47.1%) with the services provided. This satisfaction continued during the hospitalization period when the study was conducted, with patients expressing satisfaction (69; 56.7%) or high satisfaction (65; 41.4%) with the provided services (Table 2).

**Table 2:** Perceived healthcare quality at different time points

Measurement of the perceived healthcare quality	Time 1	Time 2
**Very dissatisfied**	1 (0.6%)	1 (0.6%)
**Dissatisfied**	19 (12.1%)	2 (1.3%)
**Satisfied**	65 (41.4%)	89 (56.7%)
**Very satisfied**	72 (45.9%)	65 (41.4%)

Regarding the perceived communication between physicians and patients, the participants reported that doctors answered all their questions (92; 58.6%), demonstrated understanding and empathy in finding solutions together (84; 53.5%), and actively involved patients in the decision-making process (155; 98.7%). Moreover, most patients stated that they received detailed information about the examination procedure (82; 52.2%), although a small percentage felt the information was incomplete (10; 6.4%). In addition, most surgical oncologists provided an in-depth overview (86; 54.8%). When considering the statistical differences based on patient age, it was found that patients aged 18-23 appreciated that physicians did not explain their analysis and investigations too much, in contrast to patients aged 36-41 years, who expressed complete satisfaction with the information provided (p=0.002).

The results indicate that a majority of the participants expressed high levels of satisfaction and total satisfaction with the communication between themselves and their physicians. Furthermore, they reported a high perceived quality of care. Subsequently, many participants (154; 98.1%) expressed willingness to recommend surgical oncology services to their family and friends.

## Discussion

The aim of this research was to investigate patient-physician communication in a system that would facilitate an increase in the perceived quality and recommendations of services, contributing directly to the image of a physician or an organization.

Based on the results, effective patient-physician communication was evident in the surgical oncology department. However, considering the diverse needs of cancer patients, physicians must recognize communication as a vital conduit for supporting patients and their families [5]. Furthermore, physicians should consider effective communication a core clinical skill that requires training and should be taught with the same rigor as other professional medical training [25]. In the field of surgical oncology, experts agree that several essential elements contribute to the effectiveness of communication skills training. These elements include being realistic, ensuring accuracy in medical practice, relevance to the learner because designing learning challenges will catch their attention, and appropriate challenge level according to the learner’s skill level and goals and needs. In addition, most learners study best when they feel safe and practice in a safe environment [4, 26].

In a study by Braile and Aaron [4], patient-physician communication in a surgical oncology department was classified into six main categories:

- Disease and treatment-specific communication, which covered medical histories, diagnoses and symptoms, progression and regression, prognosis, available treatments, pros and cons of the treatments, and their side effects;
- Healthcare planning topics focused on referrals, sick leave, drug prescriptions, and follow-up consultations after treatment;
- Psychological functioning/well-being addressed the psychological and well-being difficulties patients may encounter, such as insomnia, fear, anxiety, and depression;
- Daily life functioning encompassed issues related to the quality of life, living environment, social activities, and work activities;
- Coping with disease emphasized coaching strategies provided by physicians, offering support and guidance to help patients accept their condition, maintain a positive mindset, and not lose hope. Psychological counseling was also offered to patients’ relatives and friends;
- Expressions of concern and feelings revealed issues important to the patient, such as medication compliance and encouraging patients to stay active. Physicians should express understanding and empathy and clear any misunderstandings concerning the disease and the treatment during consultations.

Despite the exposure to communication and interpersonal skills during medical studies, it is difficult to prepare the medical student for the clinical challenges that may occur in surgical oncology practice, which include giving bad news, dealing with strong patient and family emotions, transitioning the patient from curative to palliative care, and discussing end-of-life issue [8]. In practice, few surgical oncology training programs offer personalized training in communication skills [8]. One successful intervention approach could include teaching residents communication skills through workshops over several days. These workshops should focus on simulation and role-play activities, using learning models designed for adult learners.

## Conclusion

Integrating patient-physician communication within a non-medical system should be considered an antecedent of perceived quality and a core element in shaping the overall image. Furthermore, it impacts patients' adherence to prescribed treatments, and physicians’ communication skills may contribute significantly to building trust. Surgical oncology is a very complex medical specialty, and it requires the constant training of physicians in communication skills. As such, communication skills should be taught to students during their medical studies and should be prioritized in the curriculum.
